# Supplementary figures and images for: Neglected Occupational Risk Factors—A Contributor to Diagnostic Delays in Lung Cancer
Source: Healthcare (Basel). 2026 Jan 1;14(1):106. doi: 10.3390/healthcare14010106 (PMC12786200; doi:10.3390/healthcare14010106)

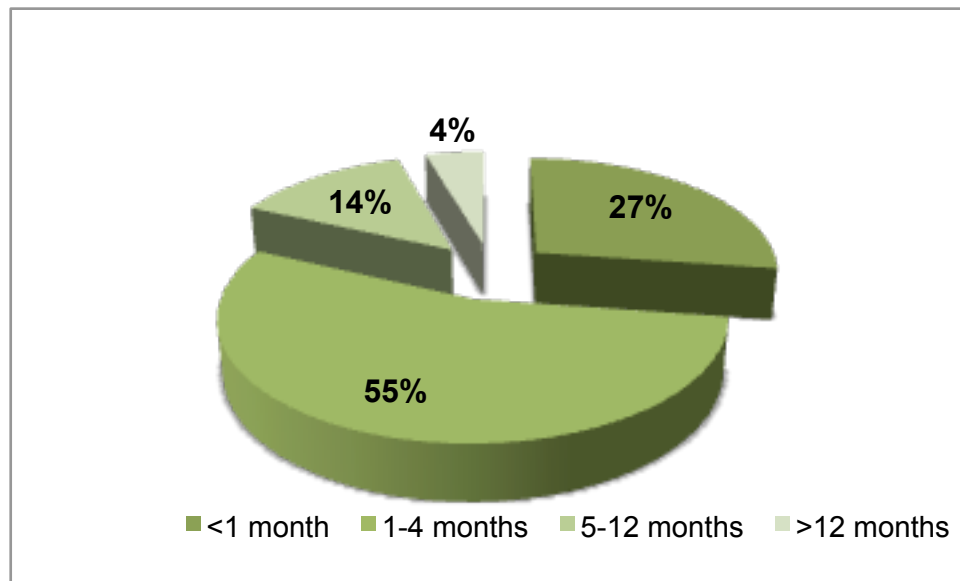

Suppl. Fig. S1. Distribution of the total interval time to diagnosis

Supplement: Supplementary file 1 [file healthcare-14-00106-s001.zip › healthcare-4059591-supplementary.pdf]
